# Male contraception: where are we going and where have we been?

**DOI:** 10.1136/bmjsrh-2019-200395

**Published:** 2019-09-19

**Authors:** John Joseph Reynolds-Wright, Richard A Anderson

**Affiliations:** MRC Centre for Reproductive Health, The Queen's Medical Research Institute, University of Edinburgh, Edinburgh, UK

**Keywords:** hormonal contraception, male contraception, review

## Abstract

Progress in developing new reversible male contraception has been slow. While the hormonal approach has been clearly shown to be capable of providing effective and reversible contraception, there remains no product available. Currently, trials of a self-administered gel combination of testosterone and the progestogen Nestorone® are under way, complementing the largely injectable methods previously investigated. Novel long-acting steroids with both androgenic and progestogenic activity are also in early clinical trials. The non-hormonal approach offers potential advantages, with potential sites of action on spermatogenesis, and sperm maturation in the epididymis or at the vas, but remains in preclinical testing. Surveys indicate the willingness of men, and their partners, to use a new male method, but they continue to lack that opportunity.

**Key messages**

- Development of male hormonal contraception has been delayed by multiple factors, but new clinical trials are closer than ever to bringing viable products to market.
- With new technologies, new potential non-hormonal targets for male contraception have been discovered.
- Developing a greater range of male methods is vital to providing holistic reproductive healthcare.

## Introduction

The Faculty of Sexual and Reproductive Healthcare vision statement^1^ focuses on patient experience, specifically that patients should have access to the full range of contraceptive options. For men, these options are currently limited to condoms and vasectomy. Both these methods have their positives—condoms continue to play a vital role in infection prevention and are a widely used, easily accessible method of contraception; likewise, vasectomy is a highly effective permanent method of contraception. However, they are not sufficient for many sexually active men who wish to have control over their fertility, and for their partners who wish to share the burden of contraception. We know that sterilisation and condoms are not sufficient for women to control their fertility, and having a greater breadth of contraceptive tools allows more couples to have better reproductive control. This review will help clinicians to understand the male contraceptives that are on the horizon and may start coming to market in the medium term.

## The life course of a sperm

Spermatogenesis begins in the brain. The hypothalamus secretes gonadotrophin-releasing hormone (GnRH), which in turn stimulates the anterior pituitary to release luteinising hormone (LH) and follicle stimulating hormone (FSH). These hormones then act on the Leydig and Sertoli cells within the testis.

Leydig cells produce testosterone, which is secreted into the bloodstream. Serum testosterone supports a wide range of male functions and is a key component of the negative feedback loop that controls GnRH and gonadotrophin production. Testicular levels of testosterone are approximately 40 times higher than in blood^2^ and support Sertoli cells in their role in spermatogenesis.

**Figure 1:**
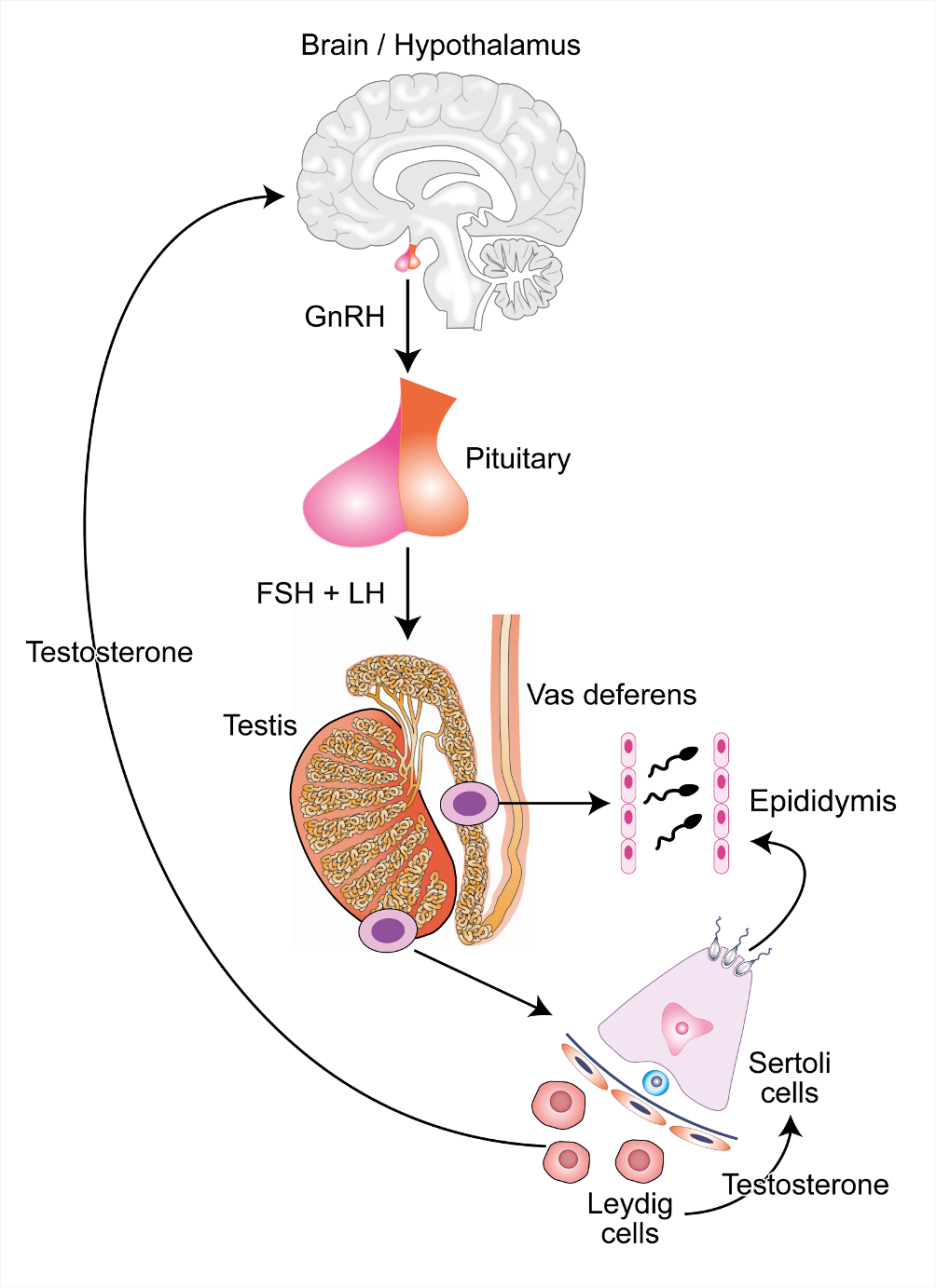
The hypothalamic-pituitary-testicular axis and its role in spermatogenesis. CCBY 4.0 Licence – John Reynolds-Wright. Image accessible via https://flic.kr/p/2hgvk5V6A. FSH, follicle stimulating hormone; GnRH, gonadotrophin-releasing hormone; LH, luteinising hormone.

After release from Sertoli cells (‘spermiation’), sperm progress through the seminiferous tubules and into the epididymis for storage, concentration and maturation to functional sperm. Finally, at the point of ejaculation, sperm are transferred from the epididymis via the vas deferens to the urethra and then out of the body (figure 1).

Potential novel male contraceptives can be divided into those based on a hormonal approach, acting via gonadotrophin suppression, and non-hormonal methods. Hormonal strategies for male contraception involve the hypothalamic-pituitary-testicular (HPT) axis, using exogenous testosterone in combination with progestogen to drive the negative feedback response and suppress GnRH, FSH and LH. Exogenous replacement of testosterone prevents hypogonadal side effects from HPT axis suppression but is not delivered at high enough concentrations to support spermatogenesis directly. Non-hormonal methods can act at any point during spermatogenesis or can target sperm maturation, detachment, motility or transport out of the testis. This includes physical methods of infiltration of the vas with a dissolvable compound to block or damage sperm during their movement along the vas.

## A brief history of hormonal male contraception

The question asked most frequently about male contraception, other than ‘When will it be ready?’, is ‘what is taking so long?’. The following summary on the history of male contraception will hopefully answer this question.

The quest to develop new forms of male contraception started at more or less the same time as the development of female hormonal contraceptive methods, but has taken a very different course. The culture shift that took place in the wake of the phenomenon of the hormonal contraceptive pill for women^3^ in 1965 led to the creation of the WHO Special Programme of Research, Development and Research Training in Human Reproduction (WHO-HRP).^3^ Within the WHO-HRP a task force for developing methods of male fertility regulation was developed. This identified possible modalities for male contraceptives through basic science research projects and multicentre landmark clinical trials of testosterone alone and in combination with progestogens.^3^ The task force also focused efforts on the development and promotion of no-scalpel vasectomy, which has become the predominant technique globally, and gossypol, which was planned as a reversible contraceptive but unfortunately had to be abandoned due to issues with toxicity and irreversibility.^3^

Over approximately a decade from the mid-1980s, the task force developed new esters of testosterone with the hope that they would be developed by industry partners for use in treatment of hypogonadal patients and as male contraceptives. However, this did not come to fruition, for reasons including concerns in industry about security of patents, possibility of litigation, perception of a ‘small market’ and competition with their existing female contraceptives. Nevertheless, landmark contraceptive efficacy studies were conducted at this time using weekly injections of testosterone enanthate. The first of these demonstrated that most men (70%) became azoospermic when administered 200 mg/week of testosterone enanthate, and couples then used this as their exclusive method of contraception for 12 months. This proved to be a highly effective contraceptive, with only 0.8 pregnancies per 100 person-years of exposure.^4^ A second study extended this to ask ‘how low does a sperm count need to go?’, and used 3 million/mL as the cut-off for entry to the efficacy phase. This increased the response rate (92% of men achieved sperm counts below this threshold and entered the efficacy phase), and again excellent efficacy was demonstrated with a Pearl Index of 1.4 (95% CI 0.4 to 3.7).^5^ Although a Pearl Index of 8.1 (95% CI 2.2 to 20.7) was achieved even in men who were not azoospermic, it was subsequently decided that <1 million/mL was the appropriate sperm count cut-off for future contraceptive efficacy studies.^6^

While these studies demonstrated that hormonal contraception using testosterone was a real possibility for men, subsequent studies have focused on a combination with progestogens, which are potent gonadotrophin suppressors in men as they are in women. In combination, they allow a much lower dose of testosterone to be used, essentially using the progestogen to do the gonadotrophin suppression, with approximately physiological doses of testosterone providing replacement for the suppressed Leydig cell function. The 1990s saw a lot of activity exploring a range of progestogens, although generally in small studies with spermatogenic suppression as the end point, rather than contraceptive efficacy.^7^ A key limitation at that time was the absence of a long-acting testosterone preparation, although testosterone pellets were used in studies, predominantly in the UK and Australia. In prototype long-acting reversible contraceptive (LARC) studies, testosterone pellets were combined with etonogestrel implants, showing excellent spermatogenic suppression,^8^ and in combination with depot medoxyprogesterone acetate (DMPA) were used in an efficacy study, with no pregnancies in 55 couples over 35.5 person-years of exposure.^9^

The development of long-acting testosterone undecanoate (TU) by Schering led to a collaborative study with Organon, which developed specific etonogestrel implants for men for a trial of that combination, confirming effective spermatogenic suppression.^10^ Unfortunately, this industry involvement was short-lived due to changing priorities in both companies, and there are no commercially funded active studies at present. In China, large studies were also conducted using TU alone.^11^ The combination approach was developed into an efficacy study by WHO and CONRAD (Contraception Research and Development network), using TU with norethisterone enanthate, both given at 8-week intervals (with the anticipation of a single combination injection being developed). This study recruited over 300 couples internationally, with 96.8% of men achieving sufficient spermatogenic suppression to enter the 1-year efficacy phase. While the trial was stopped early by a WHO review panel due to concern over side effects (despite very few men discontinuing treatment), there were just four pregnancies, giving a contraceptive efficacy of 1.59% (CI 0.6 to 4.2),^12^ thus matching hormonal female methods and substantially better than condoms, the only current reversible male method.

The essential role of GnRH in regulating reproductive function makes it an obvious target for study, since the early days of development of GnRH agonists in reproductive medicine. Both agonists and antagonists have the potential to act as contraceptives. GnRH agonists do not result in sufficient gonadotrophin suppression in men, but GnRH antagonists are very effective and there have been several studies that have investigated GnRH antagonists (with testosterone); however, these did not reduce sperm concentrations to a greater degree than testosterone-progestogen combinations.^13^ GnRH antagonists have only been available in parenteral preparations requiring serial injections or infusions, which have practicality and cost implications. New oral GnRH antagonists are becoming available, developed for the treatment of endometriosis,^14^ and may have a role to play as part of a patient-controlled contraceptive in the future.

## Current and future hormonal approaches

### Testosterone and Nestorone® (segesterone acetate) transdermal gel

A nestorone-testosterone gel (NES/T) has recently entered an international phase IIb clinical trial to establish efficacy and side effect frequency, supported by the US National Institute of Child Health and Human Development and the Population Council. Nestorone® is a novel potent progestogen with minimal activity at the androgen receptor, also licensed by the Food and Drug Administration as a combined vaginal ring for female contraception, not yet available in the UK. This is a patient-controlled, daily-dose method of combined progestogen-testosterone hormonal contraception. Minimal side effects are anticipated due to the relatively low dose of testosterone and progestogen delivered.^15^

In the development of this approach, NES/T was shown to suppress FSH and LH to less than 1 IU/L (a level consistent with achieving contraceptive effectiveness in other trials) more consistently and effectively than testosterone gel alone. There was a significant decrease in sperm concentrations in the NES/T group despite the duration of treatment being only 4 weeks, and no differences in psychosexual measures between groups or from baseline.^16^

### Kisspeptin-based HPT suppression

Kisspeptin is a compound recently discovered to play a significant role in the regulation of GnRH release by the hypothalamus. The kisspeptin-neurokinin B-dynorphin pathway acts on the hypothalamus to upregulate and downregulate the release of GnRH.^17^ It has potential to be used both in patients with hypogonadism to increase GnRH and sex steroid production, and conversely to suppress GnRH production and thus the HPT axis as a potential contraceptive,^18^ but we are not aware of any current trials related to male contraception.

### Synthetic androgen-progestogen compounds and selective androgen receptor modulators

Several chemical compounds have been developed that exhibit both androgenic and progestogenic effects and represent potential male contraceptives, combining both aspects of what is currently the leading hormonal approach and might also be useful for hypogonadism. There are various ongoing studies to develop these drugs, both as implantable and oral contraceptives. Recently, phase I trials of two oral preparations, 11-beta-methyl-19-nortestosterone dodecylcarbonate^19^ and dimethandrolone undecanoate,^20^ have been conducted showing gonadotrophin suppression and therefore promise as potential male contraceptive pills, although at a very early stage.

7alpha-methyl-19-nortestosterone (MENT) is a potent androgen which does not undergo 5-alpha reduction but is aromatised; this combination may even allow protective effects against prostate disease, while supporting sexual function and bone density.^21^ When administered as an implant (because of rapid clearance), MENT can provide good gonadotrophin and spermatogenic suppression.^22^

## Non-hormonal approaches acting on spermatogenesis

### Bisdichloroacetyldiamines

Conversion of vitamin A to retinoic acid in the testis is essential for spermatogenesis, as retinoic acid is essential for initiation of meiosis. Meiosis is a potentially attractive target for contraception, as its only function is the production of haploid gametes; thus, sufficient specificity should reduce the likelihood of off-target effects and not interfere with the endocrine function of the testis. Conversion from vitamin A is mediated by alcohol and aldehyde dehydrogenases, which can be reversibly inhibited by administration of bisdichloroacetyldiamines. To date this has been successfully used to induce azoospermia in rabbits, which reversed following withdrawal of the compound, but unfortunately in humans the drug induces an ‘antabuse’-type reaction. Further studies, including the development of agents without this side effect, are warranted as the method may induce azoospermia more rapidly than hormonal methods.^23^

### Bromodomain testis-specific protein inhibitors

Bromodomain testis-specific proteins (BRDTs) are expressed during the development of maturing spermatocytes and are important in the remodelling of cellular chromatin. A BRDT inhibitor has been shown to induce reversible infertility in mice without affecting serum testosterone levels or copulatory behaviour.^24^ Further development from this important proof of concept may be facilitated by the potential of related drugs in oncology.

### Lonidamine derivatives

Lonidamine was discovered in the 1970s and is a non-hormonal drug that is known to have antispermatogenic characteristics; however, it also had significant side effects, including testicular pain and liver dysfunction. Derivatives of lonidamine, including adjudin and gamendazole, have fewer side effects and act by causing premature detachment of spermatids from the Sertoli cells and subsequent infertility.^25^ Unfortunately, in animal studies, doses that could give effective contraception either resulted in liver side effects or irreversibility. However, further studies are in progress to modify these compounds to improve reversibility and reduce adverse effects.^26^

### Thermal treatment

Transient mild increases in testicular temperature can result in reduction in sperm count particularly when paired with a hormone administration, but is inferior to combined testosterone and progestogen treatment.^27^ There are some small communities of men practising ‘do-it-yourself’ thermal treatments, either using a hot bath method or with a modified form of underwear to create ‘artificial cryptorchidism’. There is not much current active research; however, a recent French acceptability study reported that one-third of respondents showed interest in this method as a contraceptive.^28^

## Non-hormonal approaches acting on the epididymis

The epididymis is responsible for concentrating sperm within the seminiferous fluid and conditioning the lipids and proteins on the surface of the sperm. This process takes several weeks and is essential for maturation of the sperm to improve their functionality. There are several areas currently being explored as potential sites of intervention for a male contraceptive. There are proteins, for example Eppin,^29^ expressed in the epididymis (and nowhere else in the body) that could be targeted with drugs that disrupt their functions. As these proteins are only expressed in the reproductive tract, there should theoretically be few side effects to these methods.^29^

## Non-hormonal approaches acting on the vas

### Intravasal agents

The reversible inhibition of sperm under guidance technique involves the injection of a polymer into the vas, which then acts to destabilise the cell membrane of sperm that pass through it, rendering them non-viable. As there is not a complete barrier to fluid passage along the vas, issues relating to back-pressure are avoided. The method can later be reversed either by mechanically massaging the polymerised section of the vas to dislodge the polymer or by reinjection with a dissolving agent. The technique was originally developed in India,^30^ and a related compound is currently undergoing animal trials in the USA under the brand name Vasalgel. Following successful trials in non-human primates,^31^ further preclinical studies are under way.

### Adrenoreceptor antagonists

Transport of sperm from the epididymis along the vas is mediated by smooth muscle contractility. Alpha-1-adrenoreceptor antagonists, such as tamsulosin, inhibit this contractility, and patients treated with these drugs for other reasons demonstrate reduced sperm content in semen.^32^ The contraceptive potential of these drugs has been explored only in small studies, which have shown that anejaculation can be generated at doses of 0.8 mg, but this had side effects including transient discomfort lasting up to 10 hours. At a lower dose of 0.4 mg, side effects were reduced, but higher numbers of functional sperm were found in seminal fluid. There is potential that this or a similar compound could be used as an ‘on-demand’ male contraceptive as the effect of these drugs is transient.

### Barrier methods

Barrier methods will, for the foreseeable future, have an important role in infection prevention. Novel materials and production methods are increasing the diversity of condoms available on the market with an aim to improve sensation while retaining safety and efficacy. Funding from a charitable organisation has been used to develop a self-lubricating condom that may improve pleasure and reduce discomfort, as well as reduce friction-related damage to the condom.^33^

### Microchip delivery

Novel methods of delivery of existing compounds may be possible. Microchip technology to deliver progestogen-only contraception for women is currently in development and has been used for delivery of parathyroid hormone.^34^ This format would be a truly LARC method and could overcome the difficulties in dosing frequency for testosterone alone or with a progestogen.

## Will novel male methods meet the general principles of contraceptive development?

There are five domains broadly sought after in a novel contraceptive method^35^:

1. Contraceptives must have high efficacy, both in controlled settings and in real-world use.
2. The contraceptive effect must be reversible: while there may be some utility to alternative methods of permanent contraception, safe effective methods of achieving this already exist and the gap in the market is for true, on-demand reversibility.
3. Speed of action: the time taken from initiating the contraceptive until the method offers protection from pregnancy should be as short as possible to prevent unintended pregnancy.
4. Ease of use: novel contraceptives should be simple to use, whether they are patient-initiated (pills and gels) or clinician-initiated (injections and implants), with clear ‘missed pill’ rules. This also includes issues of accessibility.
5. Safety: side effects should be minimal to reduce discontinuation, particularly with regard to secondary sex characteristics, sexual function and sexual pleasure. Non-contraceptive benefits should be maximised and should not be worse than no contraception.

It is clear that fulfilling all of these domains is closer than ever before for some of the approaches discussed above. Efficacy of currently studied methods looks to be high, with low rates of residual spermatogenesis and low rates of pregnancy. Likewise, reversibility following discontinuation appears complete and treatment modalities are familiar and easy to use (injections, gels and pills). Speed of action is where this approach falls down slightly—hormonal methods currently being investigated require several weeks of use before they can be relied on, although this is similar to vasectomy. Male hormonal methods also appear to be largely safe, being based on steroids that have been used in large numbers of people over many years, without detriment to secondary sex characteristics. Hormonal methods can have a negative effect on cholesterol, particularly decreases in high-density lipoprotein cholesterol, which may play a role in heart disease risk, but this must be interpreted with caution as it may largely reflect historical approaches based on high doses of testosterone, and risk of cardiovascular disease is complex and multifactorial. There may be positive non-contraceptive benefits of these drugs in terms of prostate function, muscle mass and bone density that will not be understood until there are longer-term, population-level data, and the use of novel steroids may increase these benefits.

## Social probabilities

Ample evidence exists to show that high proportions of men find the idea of a male contraceptive acceptable and that women would rely on their male partners to use a male contraceptive method.^36^

It is important to remember that many women rely on their male partner for contraception already—approximately 54% of women in the UK use either male sterilisation, male condom or withdrawal method as their main method of contraception.^37^

Thus, men want to engage in reproductive health, but their current options are limited compared with the range of contraceptive choices available to women. Male hormonal contraceptive methods do not currently exist on the market anywhere globally and so we cannot base our assumptions on future use of these methods on how current methods are used. Men may be attracted to male hormonal contraceptives for non-contraceptive benefits, similar to the familiar non-contraceptive benefits of female hormonal methods, and this is an aspect that can potentially be ‘engineered’ into methods based on novel steroids.

## Challenges for services

In the current research paradigm, male contraception development involves frequent semen analysis to confirm azoospermia/oligospermia prior to relying on the method as a contraceptive. During later phase studies and then as male contraceptives enter clinical practice, the focus will need to shift from intensive monitoring of sperm concentrations and more to general rules of use. However, semen analysis remains in routine clinical practice for confirming vasectomy efficacy, and home testing for spermatogenic suppression may also be a possibility with several companies developing such technology in relation to infertility.^38^

## Summary

More diverse methods of male contraceptives are needed to help meet the unmet contraceptive need of men and women everywhere. Research into these methods has been ongoing since the 1970s, but development has been hindered by a wide range of factors: challenges for funding of male methods and lack of industry involvement, disbelief that there is a real need for novel male methods, slow development of novel testosterone preparations, and the underlying physiology whereby men produce millions of sperm per day rather than releasing one oocyte per month. Fortunately, we are closer than ever to new hormonal methods coming to the market, and while non-hormonal methods seem at present more distant they have the potential to make a major contribution in the future.
