# Correction: *Male contraception: where are we going and where have we been?*

**DOI:** 10.1136/bmjsrh-2019-200395corr1

**Published:** 2020-04-17

Reynolds-Wright JJ, Anderson RA. Male contraception: where are we going and where have we been? *BMJ Sex Reprod Health* 2019;45:236–42.

There is a minor error in the paper. At present the text incorrectly reads: ‘While the trial was stopped early by a WHO review panel due to concern over side effects (despite very few men discontinuing treatment), there were just four pregnancies, giving a contraceptive efficacy of 1.59% (CI 0.6 to 4.2),^12^ thus matching hormonal female methods and substantially better than condoms, the only current reversible male method.’

The correct text should be: ‘While the trial was stopped early by a WHO review panel due to concern over side effects (despite very few men discontinuing treatment), there were just four pregnancies, *giving a Pearl index of 2.18 pregnancies per 100 person-years (95% CI, 0.82 to 5.80)*,^12^ thus matching hormonal female methods and substantially better than condoms, the only current reversible male method.

